# Underlying Neurobiological and Neurocognitive Mechanisms of Impulsivity in Risk-Taking Behaviors

**DOI:** 10.3390/brainsci10040192

**Published:** 2020-03-25

**Authors:** Melissa A. Cyders

**Affiliations:** Department of Psychology, Indiana University Purdue University Indianapolis, Indianapolis, IN 46202, USA; mcyders@iu.edu; Tel.: +1-317-274-6752

Impulsivity has been widely implicated in many maladaptive risk-taking and clinical disorders associated with such behaviors [1,2], and may be the most frequently noted criteria in the Diagnostic Statistical Manual for Mental Disorders [3] across a wide variety of disorder classes [4]. The goal of this Special Issue is to present evidence of the neurobiological and neurocognitive mechanisms underlying impulsivity. Specifically, we report evidence across a wide range of maladaptive behaviors, including alcohol and substance use, risky sexual behavior, non-suicidal self-injury, gambling, and other related behaviors. We include research spanning mathematical modeling, human neuroimaging, and behavioral and experimental approaches. We report findings measuring impulsive personality (i.e., self-report) and impulsive behavior (i.e., behavioral lab tasks), given that these complementary methods are related, but discrete [5].

D’Alessandro and colleagues present a novel joint computational modelling approach that can account for both neural and behavioral data simultaneously when studying impulsivity in neuroimaging approaches. Such computational models are key, as they allow individual-level parameters to be used to connect both behavioral and structural sources of information. Allen and colleagues report that negative urgency (a personality trait reflecting the tendency to act rashly in response to negative emotions [6]) and a behavioral measure of negative emotion response inhibition contribute independent variance to the prediction of eating disorder symptoms. The authors suggest that negative emotion response inhibition may reflect a common underlying factor of both eating disorders and non-suicidal self-injury. Steele and colleagues report on two experiments showing that the use of real and hypothetical rewards does not result in different choice patterns in experimental approaches, but that individuals are less sensitive to hypothetical delays than real delays. This study importantly suggests that it may be the reality of the delay, rather than the reward, to exhibit risk-taking behavior in the laboratory, which has implications for clinical and preclinical translational approaches. Miglin and colleagues report relationships among discrete impulsivity-related traits from the UPPS-P scale [6] and cortical thickness, highlighting an important link between sensation seeking and risk-taking that might be explained by a reduced thickness in the pericalcarine region. Glustiniani and colleagues reports findings suggesting that individuals who fail to develop a successful strategy on the Iowa Gambling task are more prone to risk. Finally, Um and colleagues reviewed the extant literature concerning the neural correlates of negative urgency and compared these correlates with those implicated in addictive disorders. Patterns of structure and function in the ventral striatum, frontal regions, and amygdala were both commonly reported across negative urgency and addictive disorders, suggesting that these conditions are likely driven by common underlying factors, which can inform development and testing of novel interventions across preclinical and clinical models.

It is my hope that this Special Issue will establish what is known concerning the underpinnings impulsivity and stimulate future work in this domain. Specifically, I hope that this program of research can begin to offer prime physiological and pharmacological targets to design and test novel interventions to reduce impulsivity-driven maladaptive behaviors and clinical disorders, especially since emerging evidence suggests that impulsivity may affect treatment outcomes [7]. There is a need to better integrate findings across preclinical and clinical literature in order to advance our ability to design and test novel treatment targets and to better advance the literature examining the neural underpinnings of impulsivity [8]. Additionally, work that assesses discrete, unidimensional measures of impulsivity [9] across the lifespan [10,11] will be best placed to advance current knowledge and treatment development.

## References

[1] Evenden J.L. (1999). Varieties of impulsivity. Psychopharmacology.

[2] Berg J.M., Latzman R.D., Bliwise N.G., Lilienfeld S.O. (2015). Parsing the Heterogeneity of Impulsivity: A Meta-Analytic Review of the Behavioral Implications of the UPPS for Psychopathology. Psychol. Assess..

[3] American Psychiatric Association (2000). Diagnostic and Statistical Manual of Mental Disorders.

[4] Um M., Hershberger A.R., Whitt Z.T., Cyders M.A. (2018). Recommendations for applying a multi-dimensional model of impulsive personality to diagnosis and treatment. Borderline Pers. Disord. Emot. Dysregulation.

[5] Cyders M.A., Coskunpinar A. (2011). Measurement of constructs using self-report and behavioral lab tasks: Is there overlap in nomothetic span and construct representation for impulsivity?. Clin. Psychol. Rev..

[6] Lynam D.R., Smith G.T., Whiteside S.P., Cyders M. (2006). The UPPS-P: Assessing Five Personality Pathways to Impulsive Behavior (Technical Report).

[7] Hershberger A., Um M., Cyders M.A. (2017). The relationship between the UPPS-P impulsive personality traits and substance use psychotherapy outcomes: A meta-analysis. Drug Alcohol Depend..

[8] Halcomb M., Argyriou E., Cyders M.A. (2019). Integrating Preclinical and Clinical Models of Negative Urgency. Front. Psychol..

[9] Wilkinson A., Townsend K., Graham T., Muurlink O. (2015). Fatal consequences: An analysis of the failed employee voice system at the Bundaberg Hospital. Asia Pac. J. Hum. Resour..

[10] Argyriou E., Um M., Carron C., Cyders M.A. (2017). Age and impulsive behavior in drug addiction: A review of past research and future directions. Pharmacol. Biochem. Behav..

[11] Liu M., Argyriou E., Cyders M.A. (2020). Developmental Considerations for Assessment and Treatment of Impulsivity in Older Adults. Current Topics in Behavioral Neurosciences.

